# Nanofertilizers for Sustainable African Agriculture: A Global Review of Agronomic Efficiency and Environmental Sustainability

**DOI:** 10.3390/nano15050390

**Published:** 2025-03-03

**Authors:** Queen Khundi, Yaqi Jiang, Yi Sun, Yukui Rui

**Affiliations:** 1 Beijing Key Laboratory of Farmland Soil Pollution Prevention and Remediation, College of Resources and Environmental Sciences, China Agricultural University, Beijing 100193, China; queenkhundi@gmail.com (Q.K.); jyq9797@163.com (Y.J.); 18732402125@163.com (Y.S.); 2 Department of Environmental Science and Engineering, University of Science and Technology of China, Hefei 230026, China; 3 China Agricultural University Professor’s Workstation of Yuhuangmiao Town, Shanghe County, Jinan 250061, China; 4 China Agricultural University Professor’s Workstation of Sunji Town, Shanghe County, Jinan 250061, China

**Keywords:** nanofertilizers, nanoparticles, nutrient-use efficiency

## Abstract

As Africa’s population continues to grow, the need for sustainable agricultural practices has intensified, sparking greater interest in nanofertilizers This review critically evaluates the agronomic efficiency and environmental sustainability of nanofertilizers in the African context. It combines existing research on nanofertilizers’ effectiveness, nutrient-use efficiency, and environmental impact. Nanofertilizers have shown a nutrient-use efficiency boost of up to 30% compared to conventional fertilizers. This review also highlights benefits such as enhanced crop yields (up to 25% increase in maize production), reduced chemical fertilizer requirements (up to 40% reduction in nitrogen application), and improved soil health. The analysis informs policy, research, and practice aimed at optimizing nanofertilizer deployment for sustainable African agriculture. The projected global population of 2.4 billion by 2050 highlights that the need for sustainable agricultural solutions has never been more important. Our review conveys an assessment of nanofertilizers’ potential contribution to Africa’s agricultural sustainability and food security.

## 1. Introduction

The world faces significant challenges in meeting the growing demand for essential commodities such as water, food and energy, alongside essential services such as healthcare, shelter and employment, while striving to reduce the number of people in need by at least 300 million [1]. The rapid depletion of natural resources due to human population growth and consumption poses a challenge to sustainable development. On the other hand, modern agriculture faces challenges, such as reliance on extra irrigation, groundwater depletion, soil deterioration, fertilizer and pesticide contamination, declining young interest in agriculture, and inefficient practices [2]. Africa is the continent with the fastest-growing population; by 2050, its population is expected to reach 2.4 billion, and it is currently facing tremendous issues in feeding its population, having shifted from being a big exporter of agricultural products to becoming a net importer over the last three decades. To cope with food scarcity, many farms in Africa use chemical fertilizers and pesticides uncontrollably, which has led to biodiversity loss [1]. However, due to high costs and financial constraints, smallholder farmers use suboptimal amounts of fertilizer, which leads to low yields [3]. Uganda serves as an example, with a target of 200 kg^−1^ of fertilizer nutrients, yet the country imports only 6.4 million tons annually. The amount of fertilizer available is substantially below the minimum suggested application objective of 50 kg ha^−1^ specified by the 2006 Abuja Declaration, an African Union attempt to encourage sustainable agriculture practices [4]. To increase food production in Africa, intensifying modern farming technology and increasing chemical fertilizer use is essential to raise crop yields and overcome these challenges [3]. To reduce global hunger and poverty, numerous agricultural processes must be improved in order to become more efficient (Zulfiqar et al., 2019). Therefore, various agricultural methods should be modified to increase agroproductivity, such as the adoption of hybrid crop types and green agrochemicals, as well as enhanced irrigation and fertilizer procedures [5]. Nanotechnology offers a powerful tool to tackle global sustainability challenges, as it provides efficient, affordable, and eco-friendly solutions for a better future [6]. Integrating nanotechnology with climate-smart agriculture can potentially lower production costs and increase yields, particularly for resource-constrained Africa’s smallholder farmers. Nanotechnology can play a crucial role in enhancing agricultural resilience to climate change, boosting productivity, and ensuring food security [7].

The usage of nanoscale fertilizers can help minimize the loss of nitrogen from leaching and runoff, as well as their rapid breakdown and volatility, which in turn, can increase nutrient availability, soil fertility, and long-term agricultural productivity [8]. Due to their high surface area-to-volume ratio and increased infiltration ability [9], nanofertilizers offer a promising alternative to conventional fertilizers. Furthermore, utilizing nanofertilizers, or “nano-biofertilizers”, can dramatically reduce environmental concerns, and they have been proved in experiments to enhance crop output by boosting the growth of seeds, nitrogen metabolic processes, photosynthesis, both carbohydrate and protein synthesis, and resilience to stress. These fertilizers require smaller volumes for application, making them easier to apply and lower in transportation costs [8].

According to Saurabh et al. (2021), nanofertilizers offer significant potential to en-hance agricultural sustainability by improving nutrient efficiency and reducing envi-ronmental impact. Research shows they can increase nutrient use efficiency by upto 30% and crop yields by 20%, potentially replacing 50% of conventional fertilizers and de-creasing their environmental footprint. This innovation, with its controlled-release mechanisms, promises to improve crop productivity and quality while addressing en-vironmental concerns, though its responsible use id essential for minimizing risks [10]. Research studies indicate that nanofertilizers can improve yields and agricultural efficiency while reducing fertilizer application rates. For example, a study conducted on rice (*Oryza sativa* L.) found that nSiO_2_ and nSiC enhanced the length of the shoots (11–37%, 6–25%) and the length of the roots (17–87%, 59–207%) of germinating rice seeds (*Oryza sativa* L.) compared with the control. Inter-root exposure to nSiO_2_, bSiO_2_, and nSiC increased aboveground catalase activity (10–55%, 31–34%, and 13–51%, respectively) as well as trace element levels [11], Meanwhile, nano enabled immunomodulation is a promising solution for agriculture due to its low doses and minimal toxicity. Nano enabled techniques can improve plant resilience to biotic and abiotic stressors, leading to more sustainable agricultural ecosystems and reduced crop losses from environmental variables [12]. Nanoscale LiFePO₄ recovered from Li batteries also has significant promise for improving agricultural quality output and reducing peanut-related allergy concerns and tackling an increasing amount of waste [13]

Needless to say, traditional nanoparticle manufacturing processes are high in energy consumption and are damaging to the environment. In contrast, employing plants to synthesize metal-based nanoparticles is comparable to the synthesis of chemicals, with the exception that chemical inhibitors are replaced by biological extracts. This approach significantly reduces the use of conventional chemicals while producing nanoparticles that are more cost-effective, less toxic, highly efficient, and environmental friendly [11].

Although nanotechnology offers significant benefits, its application in African agriculture remains limited and has not yet achieved widespread commercialization, unlike in other industries.. As a result, there is a lack of data on its adoption, making it difficult to conduct credible economic assessments of its impact on economies and society [7].

A pan-African working group on nanotechnology has been established, comprising scientists from various African countries, including Mauritius, South Africa, and Nigeria. Coordinated by the United Nations Economic Commission for Africa (UNECA), the group aims to develop a pan-African nanotech program at the master’s level, identify areas of strength in nanotech, and establish nanotechnology research clusters across Africa. Achieving these goals will enable the use of nanoscience and -technology to address key public goods, such as environmental, agricultural, and human health challenges [14].

Due to severe technological barriers, widespread use of nanofertilizers in Africa is unlikely in the near future. Even in industrialized economies, scaling up manufacturing to make nanofertilizers inexpensive remains a challenge. Furthermore, the possible environmental and human health dangers associated with their use are a considerable concern [15].

An increasing population, nutrient-poor soils, and restricted access to high-quality inputs are some of the particular agricultural problems facing Africa. Investigating alternative options is crucial since conventional fertilizers might not be enough to meet these demands in a sustainable manner. Nanofertilizers can enhance productivity in agriculture by boosting nutrient uptake and minimizing waste. Evaluating their effectiveness along with adaptability to the soil and climate of Africa may contribute to the development of more efficient agricultural programs. There is an urgent need to assess more ecologically friendly alternatives to traditional fertilizers due to their growing negative effects on the environment. Although nanofertilizers are supposed to have less influence on the environment, this review carefully evaluates these claims in the context of African ecosystems.

This review evaluates the performance and environmental sustainability of nanofertilizers in agriculture, with the purpose of offering a balanced assessment of their potential. It intends to provide important information to academics, policy makers, and farmers about whether or not nanofertilizers might help Africa’s agricultural systems become more resilient and productive.

## 2. What Are Nanofertilizers?

Nanotechnology is the science that deals with any materials or particles at the nano level (1–100 nm) in any of their dimensions and is extensively applied in various fields, such as biotechnology, engineering, medical sciences, agriculture, and the food sector [16]. In recent decades, it has achieved significant advancements, with the potential to transform the globe into an “NPs world” [11]. At the nanoscale, a nanoparticle can have three dimensions or even one dimension only. Nature has a variety of nanoparticles (NPs) with different sizes and purposes; these particles are known as natural nanoparticles [17]. Nanoparticles are particularly adaptable because of their simplicity of functionality and small dimensions, enabling them to pass into plant cell walls below the size exclusion limit (5–20 nm) and penetrate crops [18]. Certain NPs work as nanofertilizers due to their nutrient compositions, while others, such as silver (Ag)-, titanium (Ti)-, and copper (Cu)-based NPs, act as nanopesticides due to their strong antimicrobial capabilities; furthermore, nanocarriers, including carbon-based (e.g., carbon dots), polymer-based (e.g., nanocapsules, nanospheres, and nano(hydro)gels derived from natural chitosan and cellulose), and clay-based examples (e.g., mesoporous silica and montmorillonite), are utilized as nanocarriers [18]. These carriers allow for precise delivery of agrochemicals through a variety of properties and application methods; in addition, NPs have a role in plant genetic engineering by delivering genetic material that silences genes in insects and diseases like viruses [18].

Nanotechnology possesses the ability to change agriculture through increasing crop production and quality through the application of nanofertilizers; these nanofertilizers may penetrate into the cells of plant roots and leaves more effectively than conventional fertilizers, providing nutrients with greater solubility and dispersibility while preserving the soil’s physicochemical qualities [11]. Nanofertilizers are a kind of fertilizer, the nanoparticles of which are submicroscopic in size and have the ability to be encapsulated by nutrients, having a large surface area-to-volume ratio and higher mobility of nutrients compared to traditional fertilizers [19]. Common basic materials that can be nanostructured and utilized as NFs include zeolites, silver (Ag), copper (Cu), aluminum (Al), carbon (C), zinc (Zn), potassium (K), nitrogen (N), silica (Si), iron (Fe), magnesium (Mg), sodium (Na), calcium (Ca), and manganese (Mn). Banana peel [20], grape plant substrates [21], and other plant-based materials are also being used for NF preparation.

**Figure 1:**
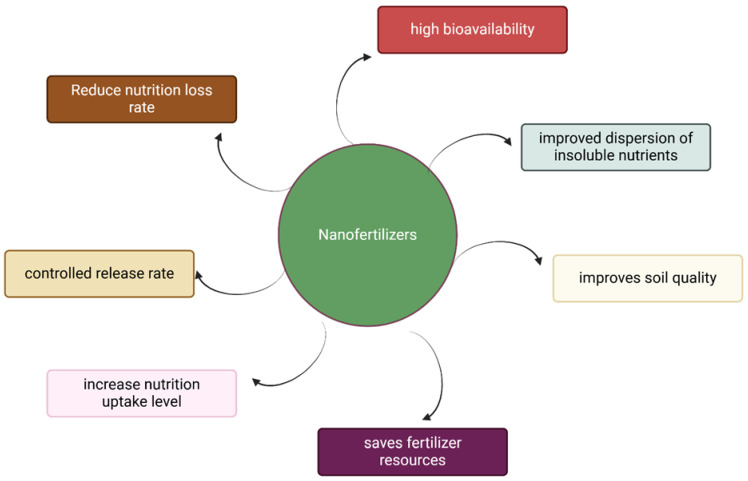
Key nanofertilizer qualities that help them be used efficiently in sustainable agriculture.

Research has shown that the adsorption of mineral nutrients is significantly influenced by negatively charged soil particles, a property known as the soil’s cation exchange capacity, which is generally larger than the anion exchange capacity in agricultural soils [22]. Application of conventional fertilizers tends to reduce the efficiency of nutrients in crops because more than 60% are lost without reaching the plant systems. Figure 1 illustrates that nanofertilizers production is a novel innovation that is safer than conventional methods, cost-effective, and ensures balanced nutrient delivery, ultimately maximizing crop yield and promoting sustainable agriculture.. It simply portrays the unique qualities of nanofertilizers, which position them as a transformative solution in sustainable agriculture, and emphasizes the distinct methods by which nanofertilizers improve nutrient efficiency and reduce ecological issues, thereby connecting broader topics of agronomic efficiency and sustainability. Precision farming is critical to the use of nanofertilizers, since it allows for controlled and site-specific nutrient delivery. This strategy improves nitrogen-use efficiency (NUE) while minimizing waste [5].

The major goal of using nanofertilizers is to manage the pace at which nutrients are delivered, thereby improving nutrient uptake efficiency to support optimal crop growth and enhance productivity [23]. This targeted nutrient delivery enhances crop yields while minimizing waste and environmental harm. By optimizing nutrient uptake, nanofertilizers also reduce soil pollution, water eutrophication, and atmospheric emissions associated with fertilizer use. To overcome these obstacles and successfully address the concerns, several strategies have been established to increase the uptake of important nutrients in bioavailable forms. These include the targeted use of micro- and macronutrient sources in conjunction with traditional fertilizers fortified with value-added ingredients [24].

**Figure 2:**
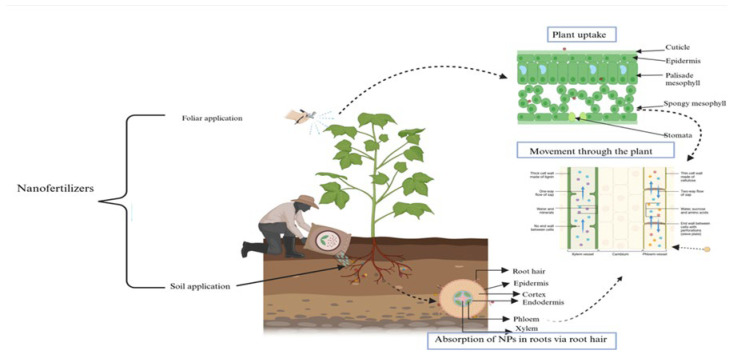
Nutrient uptake of both soil and foliar nanofertilizer applications.

Nanofertilizers can be administered to plants using both in vitro and in vivo methods. In vitro delivery strategies include soil and foliar sprays. Soil application is a highly effective method of nutrient enrichment that combines organic and inorganic fertilizers. Soil texture, PH, salt content, and salinity are important factors in determining the effectiveness of this strategy. Observations of nanofertilizers use on agricultural crops have shown that foliar application produces better outcomes than soil application [25]. Several factors influence plant uptake of nanoparticles, including nanoparticle composition, plant physiology, and the interaction of nanomaterials with the environment [26]. Nanoparticles (NPs) can penetrate plant cell walls and membranes, but their size affects entry. Cellulose limits entry to NPs between 5 and 20 nm in size, although larger NPs can create pores for entry. Once inside, NPs move through cells via endocytosis and symplastic pathways, spreading through tissues via plasmodesmata. Smaller NPs (10–50 nm) travel through symplastic routes, while larger NPs (50–200 nm) use apoplastic routes to move between cells [27]. Nutrients are transported from the soil to the root surface, following which ions are transported through membranes of root surface cells and radially transmitted into root xylem vessels, and they are dispersed in the plant’s aboveground areas [8]. The translocation process is driven by water potential [28]. Figure 2 demonstrates nutrient absorption via foliar and soil application of nanofertilizers.

Foliar application, where nutrients are adsorbed into soil particles, is regarded as one of the most important agrotechniques, with various advantages over soil application. The immediate uptake of nutrients by this application method offers quick repair of the numerous nutrient-related inadequacies that can decrease the production and quality of desirable crops [29].

This has also been observed through research that investigated the effect of micronutrient nanoparticles (MN-NPs) on common bean (*Phaseolus vulgaris* L.) in El-Menofya, Egypt. The results showed that foliar application of MN-NPs significantly improved vegetative growth, flower number, photosynthesis, and yield. The recommended concentration was 40 mg/L for optimal growth and yield improvement [30].

### 2.1. Classification of Nanofertilizers

Nanofertilizers can be made from a variety of natural and artificial materials [23]. The process of creating nanofertilizers involves combining fertilizer ingredients with nanoscale materials, such as carbon nanotubes, biopolymers, zeolites, and nanocomposites [31]. Any of these nanoscale forms can be used to deliver the primary macronutrients—nitrogen (N), phosphorus (P), and potassium (K); the secondary nutrients—magnesium (Mg), calcium (Ca), and sulfur (S); and the micronutrients—zinc (Zn), iron (Fe), manganese (Mn), copper (Cu), molybdenum (Mo), boron (B), and nickel (Ni). Any synthetic process for nanofertilizers aim to achieve objectives such as stability, efficacy, controlled dissolution rates, time-controlled release, precise concentration for targeted action, and minimal environmental toxicity, among others [31]. Nanofertilizers are classified into three types: nanoscale fertilizers, nanoscale coatings, and nanoscale additives, as illustrated in the figure below.

Due to their small particles, plants easily absorb nanomaterials compared to conventional fertilizers [32]. Nanofertilizers can be created using three methods, tailored to plant nutritional needs, to produce nanoscale coatings, additives, and nanoporous materials [8]. Nanofertilizer effectiveness depends on external elements, such as pH, soil organic matter (SOM), conductivity of electricity, texture, and mass density, as well as internal factors such as particle size and coating [32].

### 2.2. Nanoscale Additives

These are fertilizers in which nanostructured materials could be used as additives to manage fertilizer release and increase their quality (Abdalla et al., 2022). Nanomaterials replace a small amount of macroscale inputs to increase their qualities, rather than directly serving as nutrients. Nanomaterials can serve as fertilizers, micronutrients, binders, or water-retention materials for plant growth [33].

This type of fertilizer consists of nanoclays, nanosilica, or nanocarbon. Nanoscale additives improve fertilizer distribution, reduce soil compaction, promote plant growth [34], and offer insect as well as microbiological resistance for plants [33]. Nanoscale additives include nanoclay-based additives for improved fertilizer retention [34] and nanosilica additives for increasing plant yield and growth [35].

### 2.3. Nanoscale Fertilizer

Nanoscale fertilizer inputs have been produced at the nanoscale as particles or in emulsion form.Nanoscale fertilizers are nanoparticles that deliver essential nutrients to plants, including Zn, Fe, Cu, N, P, and K. Reducing the particle size of nanofertilizers improves nutrient uptake and release efficiency, providing the required quantities of nutrients to plants with reduced toxicity [33].

Nanoscale fertilizers can be characterized by increased nutrient uptake, higher crop yields, and lower application rates. Nanoscale fertilizers include nano-NPK (nitrogen, phosphorus, and potassium) fertilizers [36]. Plants can also be used to manufacture metal nanoparticles, comparable to the chemical reduction of metal ions; however, natural product extracts can replace blocking and chemical reducing agents, thereby reducing the harmful effects associated with chemical synthesis [11]. The process is easy, convenient, and environmentally friendly, reducing the use of chemical compounds while greatly increasing efficiency. Plant parts, including leaves, roots, seeds, and fruits, have been employed for this purpose [11]. The extract of a plant is added to a metal solution precursor to convert metal ions to neutral atoms then aggregate them. It is therefore utilized as a covering agent which can be used to stably synthesize metal NPs. Plants are a safer alternative to microbial synthesis strategies for producing NPs, as they do not require cell culture and are less biohazardous [11]. Additional benefits may include higher nutritional quality, a longer shelf life, and dual roles as pesticides and heavy metal scavengers [34].

### 2.4. Nano-Wrapped Fertilizers, Nanoscale Coatings, and Host Materials

Nanoscale coated fertilizers are typical fertilizers that have been coated or encapsulated with nanopolymers or loaded with nanoparticles. The coating functions as a barrier to nutrient release, helping to synchronize nutrient availability with plant growth needs and reducing environmental losses. These fertilizers are especially useful for slow-release applications, which reduce leaching and volatilization. Another excellent application of nanotechnology is the encapsulation of helpful microbes, which can significantly boost plant root growth and health. In such an application, several bacteria and fungi were used to increase the availability of phosphorus, nitrogen, and potassium in the plant root zone [33]. For instance, urea coated with nanoscale polymers can slowly release nitrogen, allowing crops to meet their nitrogen demands over time. Nanofertilizer technology has played a key role in the development of various types of fertilizers, including hybrid nanocomposites made of Cu, Fe and Zn; chitosan-based nanofertilizers; and nano-enabled fertilizers containing ZnO-NPs, FeO-NPs and MgO-NPs. ZnO-NPs have been used to coat urea granules, while nanohydroxyapatite has been utilizes as a P nanofertilizer. A lignin-clay nanohybrid has been employed as a bio-based slow-release fertilizer, and a green fertilizer with a zinc nanostructure (Zn NS) has been synthesized. Additionally, a combination of biochar and ZnO nanofertilizer has been developed, nano-rock phosphate has been used as a nano-P fertilizer and Fe3O4-urea has also been applied [23].

Metals, metalloids, anions, carbon, and biogenic nanoparticles are among the nanomaterials developed for crop development. Similarly, nanofertilizers are manufactured in diverse formulas, ranging from nanoscale macro- and micronutrients to nano-biofertilizers and nano-enabled fertilizers [31]. Nanomaterials operate exceptionally well in boosting food security, supporting the development of the food production industry [37]. Additional application areas in food processing include nanofabricated filters, nanosized adsorbents, sieves and membranes as well as catalytic agents and nanocomposite-based manufacturing equipment (via biofilm coatings) [38]. A study observed that many reviews have reviewed the potential uses of GS-MNPs in heavy metal-contaminated soil remediation, biomedical, and antimicrobial contexts. However, little research has thoroughly summarized the utilization of GS-MNPs in agriculture [11].

## 3. The Role of Nanofertilizers in Plant Growth and Resilience

### 3.1. Role of Nanoparticles in Nutrient Intake

The unique properties of nanoparticles, particularly their large proportions of surface atoms, enable superior coverage of surface areas due to their small size. Furthermore, nanoparticles may pass through cell walls of plants and animals, allowing for targeted delivery. Nanotechnologists leverage this phenomenon to achieve targeted, cellular level delivery, demonstrating superior efficacy compared to conventional methods [39].

Nanoparticles exhibit high solubility in various solvents, including water. Furthermore, nanofertilizers’ particle sizes, typically below 100 nm, allow for improved penetration in plants in the form of applied surfaces, such as soil or leaves [39]. This increased penetration ultimately improves the nutrient-uptake and -use efficiency of nanofertilizers.

Nanoparticles can be formed in two ways: top-down and bottom-up. The top-down method comprises physically breaking down bigger particles into tiny ones, while the bottom-up method uses chemical reactions to construct nanoparticles from individual atoms or molecules [40]. Meanwhile, nanofertilizers or nano-nitrogen fertilizers are made from a variety of organic and synthetic components, such as ammonium humate, ammonia, urea, peat, and plant waste. One example is when urea is coated on calcium cyanamide, resulting in a nanoformulation [41]. A new urea-coated hydroxyapatite nanoparticle fertilizer was created, allowing for slow-release nutrient delivery.This strategy addresses the challenge of efficient fertilizer delivery, particularly in African developing countries, where high fertilizer costs limit agricultural productivity . The produced nanohybrid (with a 6:1 ratio of urea to hydroxyapatite) contains 40% nitrogen, indicating the ability to sustain agricultural yields while reducing the application of urea [34].

The nitrogen-use efficiency (NUE) of nanofertilizers is approximately three times that of traditional fertilizers. Aside from that, nanofertilizers boost stress tolerance since they contain growth promoters encapsulated with nutrients within nanoscale polymers. As a result, nutrient release becomes slower and more targeted [42]. In a certain experimental study, Jiang et al. (2024) observed that soil exposure to LiFePO_4_ promoted root development in peanuts; specifically, rn-LiFePO_4_ at 50 mg kg^−1^ significantly increased the root length and root weight of peanuts by 19% and 40%, respectively [13].

### 3.2. Role of Nanoparticles in Plant Growth and Yield

The synthesis of nanofertilizers is mainly carried out to enhance crop output and quality while also increasing nutritional value; proper usage at optimal quantities can enhance crop development and yield desirable results [43]. A study has shown that applying 50% or 25% of the recommended amount of NPK nanofertilizers as a foliar treatment increased yield, starch content, nutrient-use efficiency, and harvest index in potatoes, making it the most cost-effective treatment for potato production [44]. Nevertheless, nano-growth stimulants increase seed germination and later growth stages; this was shown when soybean seed treated with nano-TiO_2_ and nano-SiO_2_ boosted nitrate reductase activity, which led to improved seed germination. An effective result was shown when both NMs were combined [45].

A study carried out in South Africa demonstrated that nanoformulated sulfate-supplemented nitrogen, phosphorus, and potassium (NPKS) fertilizer can be used to create nanofertilizers. The nanoparticles, characterized by their particle size distribution, zeta potential, and polydispersity index, were tested on maize plants under greenhouse conditions. The results showed that the nanoformulated NPKS nanofertilizers, along with inorganic NPKS, led to higher maize plant growth and higher chlorophyll contents. This suggests that incorporating sulfur nutrients into NPKS fertilizer can lead to effective maize growth and sustainable agricultural activities [46].

Another study performed in Harare, Zimbabwe, developed and deployed a slow-releasing nanocomposite fertilizer, indicating a great potential for sustainable crop production. Comparative investigations revealed that the nanocomposite fertilizer outperformed traditional NPK fertilizers in terms of nutrient release. This study demonstrated the potential of nano-based slow-releasing fertilizers in enhancing plant nutrient availability for increased growth, which could lead to more cost-effective and smarter fertilizers that increase agricultural productivity [47]. Table 1 summarizes some studies on nanofertilizer applications on cultivated plants and their impacts.

In contrast, there are also concerns concerning the use of nanofertilizers in excess. Excessive use may have major consequences in terms of nutrient toxicity that hinder crop growth [43].

### 3.3. Role of Nanoparticles in Plant Disease Control

Plant diseases, caused by factors such as severe weather, pests, viruses and nutrient defiencies, pose a major threat to global food security and economic stability [12]. Plant diseases cause 20–30% annual losses in crop production and cross-border agricultural trade. Early detection and identification of plant diseases can reduce their severity and allow for specific treatment options [58]. Nanomaterials’ distinctive optical qualities and capacity to change molecules at the nanoscale make them ideal for detecting plant diseases with great sensitivity [58]. The tiny size of nano-biosensor technology allows for stable, non-destructive, and real-time monitoring of plant health that can detect stress signals at the molecular level and translate them into visible data, allowing farmers to take early treatment measures [12].

## 4. Importance of Nanotechnology

To boost food production, the intensification of modern farming technology and increased chemical fertilizer use is essential in raising crop yields and overcoming the previously mentioned challenges [3]. Researchers have identified nanotechnology-enabled sensors as a solution for the agronomy sector to deal with climate change challenges [59].

Applying nanoscale nutrients as fertilizers can reduce nutrient losses by allowing for selective release based on time and environmental circumstances, aligning with crop absorption. Additionally, nano-nutrient fertilizers have high sorption capacities, surface areas, and controlled release of nutrients to selected areas, making them smart nutrient delivery systems [60]. Furthermore, they provide plants with enhanced resilience to biotic and abiotic stresses by releasing nutrients in a precise and timely manner, tailored to the plant’s biological and environmental needs [59].

This is evident through a South African study that investigated the use of bio-inspired calcium carbonate nanoparticles (CaCO_3_ NPs) as nanofertilizers in tomato cultivation. The research found that CaCO_3_ NPs showed moderate antifungal activity against pathogens like Cladosporium cladosporioides, Fusarium oxysporum, and Penicillium halotolerans. The study also found that different concentrations of CaCO_3_ NPs affected the gas exchange parameters, lowering water-use efficiency during vegetative and fruiting stages [61].

## 5. Nanotechnology’s Strength Regarding Environmental Challenges

Nanofertilizers have significant roles in helping plants overcome environmental stresses. The efficacy of applied nanofertilizers depends mainly on the agroecosystem conditions, including different physicochemical properties of soils, moisture, and other agroecological conditions [62].

### 5.1. Drought

The unavoidable factor that exists in many ecosystems without recognizing limits and which occurs without apparent warning is drought stress; it affects plant biomass output, quality, and energy [63].

Germination of seed is the initial step in which a crop confronts drought. Environmental conditions can cause fluctuations in germination rates, which are significant for crop management and ecology. The interaction between external elements and internal systems of seeds impacts germination under certain conditions [64].

To cope with water constraint, plants develop a variety of complex resistance and adaptation mechanisms, including physiological and biochemical responses that differ by species [63]. Nanotechnology is gaining popularity among academics across disciplines as a solution to address abiotic pressures and ensure agricultural sustainability [65]. The use of nanoparticles protects membranes, maintains water connection, and improves nutrient and water uptake, leading to a notable increase in the growth of plants under drought stress. NPs protect the photosynthetic system, accumulate osmolytes, and boost photosynthetic efficiency, while also modulating hormones, phenolics, antioxidants and gene expression to enhance plant drought resilience [66]. This was shown in a study performed in South Africa which investigated the role of hematite NPs synthesized from Aspalathus linearis in improving sorghum bicolor growth under drought stress. The study found that seed priming with 10 mg/L αFe_2_O_3_ NPs improved plant height, fresh and dry weights, chlorophyll content, anatomic structure, and nutrient distribution. Additionally, the NPs protected sorghum plants from drought-induced oxidative damage by reducing ROS formation and osmolyte accumulation [67].

Additionally, another South African study looked at the effects of iron oxide nanoparticles (FeO-NPs) on pea plants during drought stress. The results revealed that all FeO-NP treatments increased growth and yield, with the 75 ppm treatment being the most beneficial. It enhanced root length by 38% and leaf number by 24%, while decreasing malondialdehyde and hydrogen peroxide levels by 35% and 52%, respectively. Furthermore, antioxidant enzyme activity (SOD and CAT) was increased, which helped the plants battle oxidative damage [68].

Previous research has shown that Si NPs can improve plant drought tolerance. For instance, drought resistance improved in hawthorn plants treated with Si NPs, while defense-related physiological indicators varied based on drought level and Si NP concentration. Si NPs can enhance post-drought plant recovery by influencing morphophysiological features of barley plants [69].

Silicon nanoparticles exhibit enhanced efficacy due to their tiny size, which boosts solubility and mobility within plants. This leads to improved water-use efficiency through increased root growth, enhanced turgor pressure, accelerated photosynthesis rates, and elevated antioxidant enzyme activity [70]. Silicon enhances plant defenses by depositing on cell walls, increasing lodging tolerance and disease resistance through strengthened physical barriers [71]. A super-absorbent, controlled-release fertilizer containing silicon nanoparticles (SiNPs) can help minimize drought impacts by gradually releasing vital minerals and boosting soil water availability [72]. In case of seed priming, it improves germination and stress tolerance by stimulating the seed’s metabolic system, increasing vigor even in adverse conditions. This process includes breaking seed dormancy, commencing imbibition, activating enzymes, and metabolizing germination inhibitors, and seed priming restores membrane damage induced by drought stress, allowing for healthy seedling establishment [70].

A study carried out under drought-stress circumstances on soybean seed germination utilizing nano-zinc oxide and polyethylene glycol (PEG) found that nano-zinc oxide boosted the rate and percentage of germination, whereas stress reduced seed residual fresh and dry weights. These results indicate that nano-zinc oxide treatments are effective for the growth of seedlings [64].

Ahmadian et al. (2021) found that deficit irrigation reduced wheat growth and yield parameters, but nano-silica fertilizer application enhanced harvest index and yield under both irrigation conditions [73].

### 5.2. Salinity

One of the most severe abiotic pressures on the growth of plants and crop yields is soil salinity. Salinity stress may have a significant impact on world food supply. Soil salinization affects approximately 10% of the world’s total land area (950 Mha), 20% of the world’s arable land (300 Mha), and 50% of the total irrigated land (230 Mha) [74]. Nanomaterials improve the response of plants to salt by protecting photosynthesis, detoxifying ROS, and reducing osmotic and ionic stress [75]. Various nanomaterials have been employed to improve plant salt tolerance, including silica, cerium oxide, put-carbon quantum dots (put-CQD), titanium dioxide, carbon nanotubes, and nano-zinc [75].

Research suggests that zinc oxide nanoparticles can improve plant salt tolerance through multiple mechanisms, including enhanced membrane stability, reduced reactive oxygen species, controlled cell division and optimized transport of water, nutrients and carbohydrates [76]. Another study used zinc oxide nanoparticles (ZnO-NPs) to increase the Zn concentration of wheat and rice grains in saline soils. The ZnO-NPs improved growth, salt tolerance, and Zn uptake significantly when compared to other bulk Zn sources. The highest Zn concentration in grains was attained using ZnO-NPs. ZnO-NPs were more effective than other sources in saline environments [68].

Kashyap et al. (2020) performed an investigation on salt tolerance in Solanum chilense, a wild tomato cousin, by examining gene expression in seedlings exposed to sodium chloride stress. The study revealed a marked increase in the expression of genes critical for salinity tolerance, encompassing proline metabolism, ROS detoxification, and transcriptional regulation [74].

### 5.3. Waterlogging and Flooding

During waterlogging, inhibition of aerobic respiration reduces energy metabolism and hinders growth and other kinds of developmental activities, from seed germination to vegetative development and reproductive growth [77]. In times of waterlogging, NPs may have a major role in decreasing hypoxic conditions by changing metabolism and gene expression, thereby increasing plant performance [78].

During waterlogging in soybean plants, a gel-free proteomic method indicated that Al_2_O_3_ NPs outperformed ZnO and Ag to boost plant development via managing energy metabolism, causing cell death [78].

### 5.4. High Temperature

In hot summers, extreme fluctuations may impair the intermolecular linkages essential for optimum growth, impeding the growth of plants and setting of fruit. Overall, heat stress reduces the productivity of photosynthetic activities, shortening the plant life cycle and decreasing output [69,79].

Foliar application of Se NPs improved pollen germination, antioxidant enzyme activities, and productivity in sorghum plants under high-temperature stress (38/28 °C), outperforming those grown under optimal temperatures(32/22 °C). Se NPs reduced oxidant levels, shielding plants from oxidative damage [78].

ZnO nanoparticles have been demonstrated to considerably improve heat-stress tolerance in several plant species (mung bean, alfalfa, wheat, and chickpea). Zinc supplementation during heat stress can improve plant PSII efficiency, free proline levels, water relations, antioxidant enzyme activity (SOD, H_2_O_2_, MDA, and APX), and zinc ion concentrations in leaves. This can help to mitigate the detrimental effects of heat stress on plants, resulting in improved growth and photosynthesis [76].

A study undertaken by Qi et al. (2013) looked at the effects of nano-TiO_2_ on leaf photosynthesis during mild heat stress. The application of nano-TiO2 was found to have a beneficial effect on the photosynthetic processes of tomato plants, leading to increased net photosynthesis, water uptake, and transpiration, as well as reduced chlorophyll fluorescence and improved photosystem II energy regulation [80].

## 6. Overview of Nanofertilizer Application in Africa

### 6.1. Political Landscape

In Africa, 55% of land is unsuitable for sustainable agriculture. With high (16%)- to medium (13%)-quality soils, there is still limited possibility for highly profitable commercial farming due to population increase and competition from other land uses. On the other hand, the remaining 16% of low-quality soils are caused by both poor soil attributes and human-induced land degradation [81].

Fertilizer use in Africa has been low for decades, contributing to slow development in agricultural production [82]. Farmers who apply appropriate technology for their agroecological factors can achieve higher yields with the same inputs. Africa’s expanding population and limited uncultivated land require intensified farming practices. Cereal production per hectare in the region has remained constant, unlike in Asia, where the Green Revolution has greatly improved agricultural yields over decades [82].

Africa’s agricultural production landscape aligns with nanotechnology, which can boost yields while utilizing minimum resources and having a low environmental impact. Nanofertilizers have the potential to alter agricultural practices in Africa, but creating tailored nano-biofertilizers for each location and soil type is a significant problem [31]. African governments are implementing policies and programs to utilize nanotechnology, bringing together academics and industry players. Several African countries, including Kenya, Ethiopia, South Africa, Nigeria, and Zimbabwe, have undertaken nanotechnology efforts to address difficulties in key economic areas [83]

### 6.2. Compatibility of Nanofertilizers with Ecological Zones in Africa

Countries from Africa possess abundant arable resources, but their potential remains underutilized due to a shortage of skilled human capital in agriculture and agroallied sectors. Furthermore, the lack of domestic fertilizer production forces these countries to rely on expensive imported fertilizers [81,82].

The introduction of nanofertilizers to Africa has the potential to revolutionize agriculture in its diverse ecological zones. However, a crucial challenge lies in developing nano-biofertilizers that cater to the continent’s numerous agroecological regions and soil types. Ensuring the compatibility of these innovative fertilizers with Africa’s varied agroecological zones is essential for their successful adoption and sustainable impact. Africa encompasses a wide range of agroecological zones, each distinguished by distinct climate conditions, soil types, and crop preferences [31].

### 6.3. Nanotechnology Adoption in Africa

Although Africa has many potential applications for nanotechnology, adoption is still in its early stages, with most countries focusing on theoretical research and characterization [31]. The adoption of technology-specific standards can provide an indirect measure of nanotechnology progress and readiness. African countries should foster a supportive environment for nanotechnology through legislation, knowledge generation, industry, and market development [83]

The Sub-Saharan region faces significant food security challenges, yet its nanotechnology infrastructure is severely underdeveloped. South Africa stands out with cutting-edge nanoscience centers developing innovative smart-release composites to minimize fertilizer usage, while Kenya and Nigeria have identified agriculture as a national priority, but financial constraints hinder the implementation of proposed initiatives [84].

A Science-Policy Brief for the Multistakeholder Forum on Science, Technology and Innovation for the Sustainable Development Goals (SDGs) highlighted that Sub-Saharan Africa’s (SSA’s) relatively low fertilizer usage presents a unique opportunity for nanofertilizers to emerge as a viable alternative to conventional fertilizers. The underdeveloped support system for fertilizers in SSA offers a “green window” for innovation. However, the successful adoption of nanofertilizers requires a holistic approach, addressing not only technological advancements but also institutional, infrastructural, and economic needs. Agricultural practices in SSA are deeply rooted in community culture, traditions, and longstanding investments in established techniques. These sociocultural factors significantly influence decision making regarding the adoption of new technologies, highlighting the importance of considering these aspects when introducing nanofertilizers to the region [85].

### 6.4. Challenges and Future Perspective

Nanotechnology is a promising tool for developing countries to bridge technological gaps (United Nations Economic Commission for Africa, 2020). Better still, nanofertilizers have the potential to increase the quality of soil and agricultural output in Africa, but their implementation faces hurdles. The continent’s underdeveloped nanotechnology environment poses a significant challenge to its use in agriculture [31].

Nanotechnology demands modern microscopes and room facilities should be clean, making it a capital-intensive field. Despite having applicable policies and objectives, many African countries lack sufficient financial, physical, and human resources to perform nanotechnology research [83]. Studies by Arora et al. (2024) highlight that the successful implementation of next-generation fertilizers requires a multidisciplinary approach, combining nanotechnology, biotechnology, and agronomy to optimize nutrient delivery and environmental sustainability [86].

On a brighter note, African countries have embraced nanotechnology curricula in their educational institutions, providing expertise for innovative development [31]. Nanotechnology is taught as a separate topic in chemistry, physics, and biology schools. A lack of qualified specialists to teach students about nanotechnology is a major challenge. Africa can tap into the knowledge and nanotechnology capabilities of its diaspora to foster innovation and promote sustainable development in multiple sectors. The continent would benefit from encouraging professionals to visit their home countries as part of capacity-building activities. This can be adopted from the soccer paradigm, in which professional players from other continents are asked to serve their native countries, providing valuable insights [83]. Saurabh et al. (2024) emphasize that interdisciplinary collaborations among scientists, agricultural stakeholders, and regulators could help address these gaps by facilitating knowledge exchange and joint research projects [10].

## 7. Negative Impacts of Nanofertilizers on Soil Health, Animal Welfare, Human Safety, and the Environment

### 7.1. Soil Health

Soil is the primary recipient of nanoparticles, and understanding their behavior and risks in arable ecosystems is a pressing concern [87]. Soils are home to a vast range of microbes [88]. Soil microbial diversity plays a crucial role in nutrient cycling, contaminant removal, and overall soil health. However, nanomaterials in agricultural systems can accumulate in soil and impact microbial communities [89]. As a natural matrix rich in native nanoparticles, the introduction of artificial nanoparticles can significantly impact soil dynamics, potentially leading to persistence, accumulation, and unforeseen consequences [87]. Certain soil microorganisms, including N_2_-fixing bacteria, organic-matter decomposers, and methanogenic bacteria, are sensitive to nanoparticle (NP) presence. Specifically, TiO_2_-NPs (2 mg/g) and ZnO-NPs (0.5 mg/g) can negatively impact these microorganisms [6]. The impact of nanofertilizers on soil microbial communities varies depending on soil type and physicochemical conditions. Soil pH is a critical factor influencing nanoparticle behavior. For instance, zinc oxide nanoparticles dissolve into ionic species more quickly at low pH levels, whereas they tend to aggregate at higher pH levels [90].

A study investigated the environmental impacts of TiO_2_ and ZnO ENPs on soil bacterial communities. The results showed that both nano-TiO_2_ and nano-ZnO reduced microbial biomass and diversity, altering the composition of the soil bacterial community. The effect of nano-ZnO was stronger than that of nano-TiO_2_, suggesting that nanoparticulate metal oxides may negatively impact soil bacterial communities [91].

Silver nanoparticles (AgNPs) have a considerable toxic effect on enzyme activity at very low doses. AgNPs were also shown to have a concentration-dependent effect on earthworm development rates and populations, with extended exposure to high concentrations potentially resulting in population decrease or perhaps extinction [88].

In addition, in a microcosm experiment, CuO nanoparticles were found to significantly increase soil N_2_O emissions by 92.7%, potentially posing environmental risks due to their inhibition of the denitrification process and changes in related functional genes, e.g., nirK and nosZ [92].

In another model experiment with Californian red worms (*Eisenia foetida*), aluminum oxide nanoparticles were shown to negatively impact soil biocenosis and intestinal microflora in a dose-dependent manner. The application of AI_2_O_3_ NPs caused significant reductions in soil microorganism counts, with soil fungal, nitrogen-fixing bacteria, and starch-and-ammonia bacteria populations decreasing by up to 85.7%. Additionally, the number of microorganisms in the intestines of *E. foetida* decreased by up to 73.3%, indicating the toxic effects of AI_2_O_3_ NPs on both soil and intestinal ecosystems, potentially leading to soil degradation and reduced fertility [93].

### 7.2. Plants

Internalized nanoparticles (NPs) can have a significant impact on plant physiology by inducing oxidative stress, which causes the production of reactive oxygen species (ROS) and oxidative damage that can lead to cell death, protein degradation, DNA damage, and lipid peroxidation [27].

This was evident in a study that investigated the physiological mechanisms of magnetic (Fe_3_O_4_) nanoparticles in mitigating the toxicity of heavy metals in wheat seedlings. The results showed that heavy metals significantly affected seedling growth, with Cd and Cu causing significant decreases in root length and shoot length. The addition of nano-Fe_3_O_4_ significantly decreased growth inhibition and activated protective mechanisms to reduce oxidative stress. The reducing effects depended on increased enzyme activity and decreased MDA contents [94].

While the application of Fe/SiO_2_ NPs generally promoted plant growth, a concentration of 25 mg kg^−1^ of nano-Fe/SiO_2_ had a negative impact on barley, reducing shoot length. This suggests that higher concentrations of nano-Fe/SiO_2_ may have detrimental effects on certain plant growth parameters [95].

Another two-season field study in Egypt looked at the impact of a nanoparticle-based fertilizer (Hyper Feed Amino NPs) on wheat productivity and genotoxicity compared to conventional mineral fertilizers.Although NPK nanoparticles enhanced yield, they also induced chromosomal abnormalities in root-tip cells, including disrupted micronuclei formation, chromosome fragmentation and irregular mitosis . The greatest NPK NP treatment resulted in a considerable increase in aberrant cell frequency (35.79% in Sids 12 and 38.98% in Al-Rasheed), indicating possible genotoxic hazards. These findings indicate that while nanoparticle fertilizers increase productivity, they may also interfere with normal cellular functioning in plants [96]. Furthermore, Karunakaran et al. (2017) found that iron oxide and manganese oxide nanoparticles and microparticles applied on Chlorella pyrenoidosa showed distinct toxicity patterns, with MnO microparticles being highly toxic due to electrostatic interactions and greater hydrophilicity [97]. In another study, MnOx NPs were evaluated for their effects on the germination and growth of lettuce sativa seeds in a hydroponic system. While high concentrations of MnOx NPs (50 mg L^−1^) slightly reduced germination rates from 84% to 63%, this decrease was not statistically significant. Notably, MnOx NPs significantly enhanced seedling development by promoting root elongation [98].

The toxicity mechanisms of copper nanoparticles (CuNPs) have been extensively studied in animal and human systems, but their photoxicity, particularly in plants, is not well understood [99]. A study investigated the effects of iron (Fe/Fe_3_O_4_) and copper (Cu/CuO) nanoparticles and their ionic counterparts on hydroponically grown lettuce. The results showed that iron nanoparticles and ions had a negligible impact on plant physiology. In contrast, copper nanoparticles and ions significantly reduced water content, root length, and dry biomass. Notably, Cu/CuO NP treatments led to substantial Cu accumulation in roots, increased catalase activity, and decreased ascorbate peroxidase activity. Furthermore, Cu NO altered the nutritional profile of lettuce, increasing Cu, AI, and S levels while decreasing Mn, P, Ca, and Mg [100]. Additionally, a study evaluated the phytotoxic effects of CuO NPs on maize and rice. The study revealed that CuO NPs did not impact seed germination at any of the tested concentrations. However, at a high concentration of 2000 mg L^−1^, CuO NPs significantly inhibited root growth in both crops (95.73% in maize and 97.28% in rice). In addition, maize shoot length was reduced by 30.98% at this concentration [99].

### 7.3. Human and Animal Safety

NPs have a toxic impact on plant development, elongation of roots and shoots, biomass yield, chlorophyll content, photosynthetic processes, and enhanced activity of catalase and peroxidase enzymes in treated plants’ roots. Because of their small size, nanoparticles can easily permeate the environment and pose eco-toxicological risks [101].

Animals in both land and water environments are vulnerable to nanoparticle (NP) toxicity. A wide range of species, including fish, mammals, amphibians, earthworms, invertebrates, plankton, and algae, are being severely affected by NP toxicity due to their widespread presence in the environment [102].

Toxicology studies suggest potential adverse effects of nanoparticles on the immune system, oxidative stress, and diseases like cancer, but these effects typically occur at high doses. It is unclear if environmental or occupational exposure could lead to such harm. Additionally, nano-encapsulated pesticides may pose risks of soil contamination, environmental spread, and entry into the food chain, potentially affecting a wide geographic area [103].

Chronic toxicity can occur when nano-heavy metals easily penetrate the body, accumulating in vital organs and potentially causing organ failure. Additionally, plants can absorb these nano-chemicals, storing them in edible parts like leaves, fruits, and tubers, which can lead to toxic effects in humans who consume them [104].

A study by researchers at Missouri University in the USA found that nanoparticles can remain as residues in fruits, allowing them to enter the human body and potentially reach organs like the spleen, brain, liver, and heart via the bloodstream and lymphatic system. The study also showed that these residues are difficult to remove with standard rinsing [17].

Wang et al. (2012) found that CeO_2_-NPs were absorbed by tomato roots and transported to shoots and fruits. Although 10 mg of L^−1^ CeO_2_-NPs slightly enhanced plant development, the buildup of Ce increased in edible tissue (fruits), posing a health risk.

In a study on zebrafish larvae (Danio rerio), exposure to AI_2_O_3_-NPs and AI(III) led to significant genotoxic effects, with DNA damage being more severe in larvae exposed to AI(III) (41% tail DNA) compared to AI_2_O_3_-NPs (21.8% tail DNA).The LC50 values of 3.26 ± 0.38 mg/L and 130.19 ± 5.59 mg/L suggest that these substances are toxic and may pose environmental and health risks. Moreover, stress-associated genes, including mt2, were significantly induced, highlighting the adverse biological effects of AI_2_O_3_-NPs and AI(III) on aquatic organisms [105]. Using the innovative tank diving test, researchers assessed anxiety-like behavior in zebrafish. Exposure to SiO_2_ NPs or reserpine induced depression-like behavior in adult zebrafish, characterized by reduced exploratory activity and locomotion and altered swimming patterns. Immunohistochemical analysis revealed changes in neurotransmitter levels, suggesting that SiO_2_ NPs may trigger depressive effects in the neurological system [106].

Another study found that silver nanoparticles (AgNPs) caused size-dependent cytotoxicity in human lung cells due to significant silver release. In vitro studies revealed that AgNPs induced various toxicities, including inflammation, genotoxicity, and cytotoxicity, depending on particle size. Similarly, small titanium dioxide (TiO_2_) nanoparticles (<10 nm) showed immune toxicity in rat’s lungs, with different sizes exhibiting varying levels of toxicity [88].

Furthermore, recent studies on PbO/PbO_2_ NPs (PbO_x_ NPs) in maize have demonstrated significant phytotoxic effects, including alterations in seed germination, root elongation, and overall growth. The effects were dose- and size-dependent, with the nanoparticles impacting water uptake, which in turn influenced maize growth and biomass production. PbO_x_ NPs also significantly affected the nutrient contents in the roots and shoots of maize and led to bioaccumulation of lead in maize cell organelles. These findings highlight the potential threat of PbO_x_ NPs to agricultural productivity and food security, with implications for human dietary health [107].

People who work in agriculture and those residing near farms may be at risk of exposure to nanofertilizers during the application process [10]. This includes engineers, scientists, and technicians during research and production. Handling raw materials; operating equipment; and characterizing, packing, and transporting nanomaterials are key means of exposure [88].

The harmful effects of nanoparticles vary according to their shape, size, and characteristics. Certain metal NPs, such as iron, platinum, silver, and gold, as well as metal oxide NPs like ZnO, Fe_3_O_4_, and TiO_2_, utilized in a variety of industries, can be hazardous to human health. When these nanoparticles come into contact with cells, they destroy protein, DNA, and membranes, causing a dramatic reduction in plant development. These nanoparticles can also penetrate the bloodstream and reach crucial organs of humans, where they can cause severe poisoning [66].

### 7.4. Environmental Concerns

Environmental nanoparticles (NPs) undergo aging processes, including disaggregation, chemical transformation, and aggregation, which alter their properties and behavior. Nanoparticles (NPs) can enter the environment at various stages of their life cycle. Three main emission scenarios are considered: release during use, release during production, as well as release after disposal of NP-containing products through waste handling [108]. Uncontrolled and widespread use of nanoparticles, including conventional and engineered NPs, can lead to environmental accumulation, causing harmful effects on soil, microorganisms, and plant ecosystems [109].

The use of nanotechnology-based products for environmental applications is growing, leading to increased releases of engineered nanomaterials (ENMs) into the environment through applications like groundwater remediation, nanopesticides, and nanofertilizers [110]. When nanoparticles enter the environment, they interact with surrounding air, water, and soil, triggering changes to their surface characteristics. This, in turn, can cause the particles to clump together or undergo shifts in their electrical charge and other surface attributes [111]. Furthermore, the overproduction of reactive oxygen species (ROS) and resulting oxidative stress is a primary mechanism of nanotoxicity induced by nanomaterials. ROS generation depends on the physical and chemical properties of nanoparticles, such as size, shape, oxidation status, surface area, and coating, as well as environmental factors. These properties influence the level of ROS production and subsequent nanotoxicity [112].

Therefore, during nanofarming operations, there are numerous potential sources of nanopollution. Excessive use of nanomaterials before, during, and after cultivation can lead to this outcome [113]. Nanomaterials (NMs) can dissolve to varying extents, influencing their environmental behavior, effects, and fate. Dissolution can be controlled by particle shape or dissolved species. When NMs dissolve in water, molecules or ions can react with media components, forming complexes or residues [114]. Releasing nanoparticles into the environment poses significant risks, including toxicity to plants, imbalances in soil ecosystems, and potential harm to humans and animals, with long-lasting effects on the environment [27]. Additionally, nanopollutants can include materials such as nanoglass, nanofibers, nanopolymers, nanobiologicals and metal oxide nanoparticles. Glass nanoparticles can be produced from waste windshield glass and pose a significant risk to both the environment and human health. These glass nanoparticles can impede wheat seed germination, diminish root and shoot length, and decrease chlorophyll content [113]. There are two processes that produce hazardous NPs, namely, chemical and physical operations, unlike the NPs produced by biological methods [8]. Engineered nanomaterials pose significant risks to soil organisms.A range of NMs, including CNTs, carbon-based NPs such as graphene oxides, C70 ; metal oxide nanoparticles like CuO, TiO_2_ and ZnO; and nanoscale zero-valent iron (nZVI), were under investigation for their effects on soil organisms. ZnO was shown to be the most poisonous, with 100% mortality in *E. coli*, *B. subtilis*, and *P. fluorescens*. However, CuO was found to be more harmful than ZnO to *P. chlororaphis*, which is a helpful bacterium [101].

## 8. Conclusions

Nanofertilizers hold tremendous promise for revolutionizing African agriculture. By boosting nutrient uptake, minimizing waste, and reducing environmental harm, these innovative products could be a breakthrough for farmers on the continent. In Africa, where soil fertility is declining and resources are scarce, nanofertilizers could be very transformative. However, despite their potential, several obstacles are hindering their widespread adoption. These include a lack of research, prohibitively high production costs, and regulatory challenges.

To truly transform agriculture in Africa, we need to bring together the best of nanotechnology, local farming expertise, and smart policy making. Governments, universities, and businesses must join forces to create practical solutions tailored to Africa’s unique agricultural environments. Looking ahead, it is crucial that we invest in research to understand the long-term impact of nanofertilizers on soil health, crop yields, and the environment and investigate the economic viability of nanofertilizers for smallholder farmers in Africa, including cost–benefit analysis, affordability, and accessibility. Only then can we confidently integrate these innovative tools into African farming systems for generations to come.

## Figures and Tables

**Table 1:** List of some studies on nanofertilizers applied on cultivated plants and their impacts.

Cultivated Plant	Nanofertilizer Applied	Dose Applied	Parameters of Plant Studies	Refs.
Tomato (*Solanum lycopersicum*)	Iron nanoparticles (FeNPs)	Soil (0, 100, 300 mg/L), foliar (0, 100 mg/L)	Plant growth: shoot length (42%), root length (66%), number of branches (45%), number of leaves (173%), fruit weight (24%); photosynthetic pigments (carotenoids, lycopene); biochemical properties (protein, phenolic, flavonoid content); antioxidant activity; Cd accumulation reduction	[48]
Tomato (*Solanum lycopersicum*)	Nano-hydroxyapatite (PN1, PN2)	0.12 g (PN1) and 0.23 g (PN2) per 5 kg pot	Yield parameters: fruit number (50% with CaP2), average fruit weight (29% with CaP2), fruit yield (91% with CaP2); juice content; antioxidant activity; fruit volume; shoot fresh and dry weight; root length; soil texture influence on phosphorus efficiency	[49]
Tomato (*Solanum lycopersicum*)	Fe: Fe_3_O_4_@HA@GO-Cs (graphene oxide–chitosan-coated humic acid@Fe_3_O_4_ nanoparticles), Zn: AZP@GO-Cs (graphene oxide–chitosan-coated ammonium zinc phosphate)	Slow-release fertilizer with NPK, Fe, and Zn	Growth parameters: improved nutrient uptake in shoots; nitrogen (44%), phosphorus (+66%), potassium (46%), iron (75%), and zinc (74%); soil salinity and texture effects on plant performance	[50]
Soybean (G. max (L.) Merrill)	Copper oxide nanoparticles (CuO NPs)	20 nm @ 1 mg/kg, 20 nm @ 10 mg/kg, 50 nm @ 1 mg/kg, 50 nm @ 10 mg/kg	Fresh biomass (significantly increased by 50 nm CuO NPs at 10 mg/kg) Increase in activities of N assimilation-associated enzymes Nitrogenous compounds (nitrates, proteins, amino acids) and Cu contents in shoots, roots, and soils increased	[51]
Cowpea (*Vigna unguiculata*)	Copper (Cu) nanofertilizer	Foliar: 20, 40, 60, 80 ppm; soil: 25, 50, 75, 100 mg/kg	Morphological attributes (average increments: 69.09% in soil, 64.91% in foliar application); chlorophyll content (average increments: 73% in soil, 68% in foliar application); antioxidant concentration (average increments: 81% in soil, 52% in foliar application); detrimental impacts observed at higher nanofertilizer concentrations; X-ray diffraction, SEM, and FTIR used to characterize nanoparticles	[52]
Cassava (*Manihot esculenta*)	Zinc oxide (ZnO) and cadmium-doped zinc oxide (CdO-ZnO) nanoparticles	ZnO NPs and CdO-ZnO NPs in experimental treatments	Macronutrients and micronutrients enhanced in low marginal soils; improved root development, photosynthetic rate, and nitrogen-use efficiency; yield increase in cassava tubers: 23.91% (ZnO NPs), 28.91% (CdO-ZnO NPs), 18.67% (chemical fertilizer), and 14.58% (control); improved plant height and number of branches	[53]
Cucumber (*Cucumis sativus*), broccoli (*Brassica oleracea*), okra (*Abelmoschus esculentus*)	Biochar-based nanocomposite (BNF)	37.5 kg/ha	Germination parameters improved: time for 50% germination: 3.4 days (cucumber), 3.3 days (broccoli), 4.4 days (okra) ; germination index: 3.1 (cucumber), 3.5 (broccoli), 2.4 (okra) ; final germination percentage: 91.7% (cucumber), 100% (broccoli), 83.3% (okra); enhanced shoot and root length for all plants ; improved water absorbance (68%), equilibrium water content (78.97%), and swelling ratio (3.64 g g^−1^) ; long-term slow release of nitrogen, potassium, and phosphorus ; enhanced soil quality and organic matter provision	[54]
Wheat (*Triticum aestivum*)	Nano-carbon synergist, nano-calcium carbonate synergist, composite nano-synergist	CK: compound fertilizer - T1: compound fertilizer + 0.3% nano-carbon synergist - T2: compound fertilizer + 0.3% nano-calcium carbonate synergist - T3: compound fertilizer + 0.3% composite nano-synergist - T4–T6: 70% compound fertilizer + respective nano-synergists	Increased nitrogen (N) accumulation: 40–50% (T1), 30–40% (T2), 55–65% (T3), 20–30% (T4), 15–20% (T5), 30–40% (T6) compared to CK; enhanced nitrogen-use efficiency (NUE): 12–19% (T1), 9–18% (T2), 16–22% (T3), 5–17% (T4), 4–16% (T5), 10–20% (T6); significantly increased expression of nitrogen transport and metabolism-related genes: TaNRT2.2, TaNRT2.3, TaGS1, TaGS2	[55]
Chickweed (*Stellaria media*)	Nano-fulvic-like acid fertilizer (recycled from waste milk)	Not specified	Improved yield and root elongation in pot experiments; slow-release properties of the fertilizer ensured prolonged nutrient availability	[56]
Rock melon (*Cucumis melo*)	Nanoemulsion-based MARDI nanofertilizer	Varying application frequencies: T1 = 0, T2 = 2, T3 = 4, T4 = 6, T5 = 8 times (foliar treatment)	Plant height increased by 15.07% (T3) Stem diameter increased by 7.8% (T3) Relative chlorophyll content and leaf area increased by 41.5% (T3) Chlorophyll a increased by 21.1% (T5) Chlorophyll b and carotenoid increased by 24.99% (T4) Dry-weight biomass increased compared to control	[57]

## Data Availability

The article describes a study that used no data.
